# Preparation of Transdermal Patch Containing Selenium Nanoparticles Loaded with Doxycycline and Evaluation of Skin Wound Healing in a Rat Model

**DOI:** 10.3390/ph15111381

**Published:** 2022-11-10

**Authors:** Dhiya Altememy, Moosa Javdani, Pegah Khosravian, Anita Khosravi, Elham Moghtadaei Khorasgani

**Affiliations:** 1 Department of Pharmaceutics, College of Pharmacy, Al-Zahra University for Women, Karbala 56001, Iraq; 2 Veterinary Surgery Section, Department of Clinical Sciences, Faculty of Veterinary Medicine, Shahrekord University, Shahrekord 115, Iran; 3 Medical Plants Research Center, Basic Health Sciences Institute, Shahrekord University of Medical Sciences, Shahrekord 115, Iran; 4 Department of Clinical Sciences, Faculty of Veterinary Medicine, Shahrekord University, Shahrekord 115, Iran; 5 Pathobiology Department, Shahrekord Branch, Islamic Azad University, Shahrekord 115, Iran

**Keywords:** chitosan, doxycycline, selenium nanoparticle, skin wound healing, rat

## Abstract

The present study aimed to prepare and evaluate a controlled-release system based on a chitosan scaffold containing selenium nanoparticles loaded with doxycycline. Its topical application in skin wound healing in rats was investigated. Therefore, 80 female rats were used and, after creating experimental skin defects on their back, were randomly divided into four equal groups: the control group without any therapeutic intervention; the second group received a chitosan transdermal patch (Ch); the third group received chitosan transdermal patch loaded with selenium nanoparticles (ChSeN), and the last group received chitosan transdermal patch containing selenium nanoparticle loaded by doxycycline (ChSeND). Morphological and structural characteristics of the synthesized patches were evaluated, and in addition to measuring the skin wound area on days 3, 7, and 21, a histopathological examination was performed. On the third day of the study, less hemorrhage and inflammation and more neo-vascularization were seen in the ChSeND group. Moreover, on day 7, less inflammation and collagen formation were recorded in the ChSeN and ChSeND groups than in the other groups. At the same time, more neo-vascularization and re-epithelialization were seen in the ChSeND group on days 7 and 21. In addition, on day 21 of the study, the most collagen formation was in this group. Examination of the wound area also showed that the lowest area belonged to the ChSeND group. The results showed that the simultaneous presence of selenium nanoparticles and doxycycline in the ChSeND group provided the best repair compared to the control, Ch and ChSeN groups.

## 1. Introduction

Wound care is an important category in wound healing management. Although therapeutic interventions for wound management have a long history, the use of tissue engineering plays an important role in this regard. New therapeutic strategies in wound management are based on biological processes and natural wound-healing mechanisms. Additionally, facilitating drug delivery to the affected area, synthesis of scaffolding suitable for tissue and skin growth and creating a protective coating on wounds are some of the principles considered in wound tissue engineering [1]. One of the advances in this field is the synthesis of transdermal patches. These patches are adhesive patches that are placed on the skin to deliver a specific dose of the drug through the skin into the bloodstream. The main purpose of the supply of these drug forms is the local release of the drug to increase the presence and absorption of the drug in the desired location, which can be designed as a control-release drug system [2].

Chitosan is one of the materials used as a scaffold in these drug delivery systems. Chitosan has antimicrobial and antioxidant properties, especially antifungal, and stimulates the immune response and heals wounds, and due to the cationic properties of chitosan, it can be used as a coagulant in wounds [3,4]. Chitosan improves wound healing, especially during the proliferative stage and matrix formation, and stimulates the migration of inflammatory cells [5]. Chitosan transdermal patches retain water in the underlying layers of the skin and cause cell fluidity [6]. In addition to the positive effects of chitosan on wound healing, the healing process can be further improved by loading various compounds and drugs on chitosan. Therefore, the present study loaded doxycycline and selenium nanoparticles onto the chitosan scaffold to evaluate skin wound healing.

Doxycycline is a chemically modified semi-synthetic tetracycline [7]. Tetracyclines inhibit matrix metalloproteases, a family of zinc-dependent proteases involved in tissue regeneration, inflammation, and neo-vascularization [8]. Doxycycline also inhibits the conversion of proMMP to active matrix metalloproteinases by inhibiting reactive oxygen species [9,10]. The topical administration of doxycycline significantly accelerates its effect, and no side effects have been observed with the topical use of doxycycline. Therefore, topical doxycycline may be a suitable alternative to oral doxycycline [11]. Another mechanism of action of tetracyclines is the inhibition of protein synthesis by binding to bacterial ribosomes [8].

Including nanoparticles in various pharmaceutical materials may increase the beneficial effects of that drug formulation and enable better dose control [12]. Many antioxidant enzymes and proteins used in active centers contain selenium and play a key role in reducing oxidative stress in the body. Selenium nanoparticles have attracted more attention due to their excellent biological activity and lower toxicity [13]. Biosynthesized selenium nanoparticles from extremophilic Actinobacterium have been shown to have antioxidant and antiviral properties and help wound healing [14]. Different mechanisms for the beneficial effect of selenium nanoparticles on wound healing have been mentioned in various studies, and it seems that combining this nanoparticle with a chitosan scaffold will bring better healing effects.

According to the above, a controlled-release system based on a chitosan scaffold was constructed on which doxycycline and selenium nanoparticles were loaded in the present study. While examining the synthesized patches’ physical structure, swelling and adhesion strength and determining the release of loaded selenium nanoparticles, the effectiveness of these patches in the skin wound healing process in rats was evaluated.

## 2. Results

### 2.1. Evaluation of Morphological Properties of Prepared Nanoparticles Using FE-SEM

**Figure 1:**
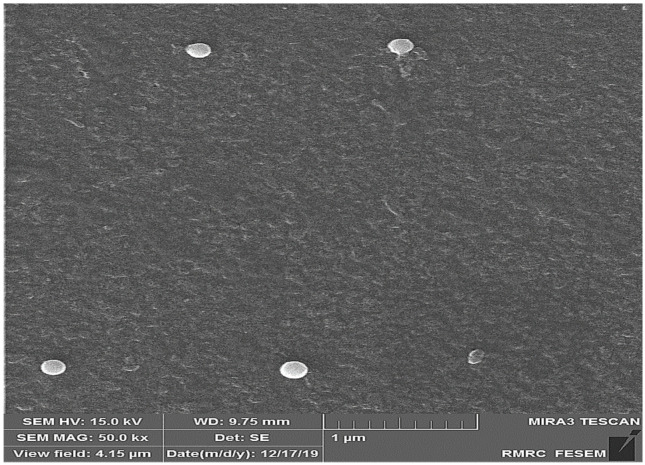
FE-SEM image of SeN. According to the picture, the size of nanoparticles is about 200 nm.

The results of the study of SeN using FE-SEM showed that the shape of the particles was spherical and uniformly distributed, and their size was about 200 nm (Figure 1).

### 2.2. Results of Particle Size, Particle Distribution and Zeta Potential

**Figure 2:**
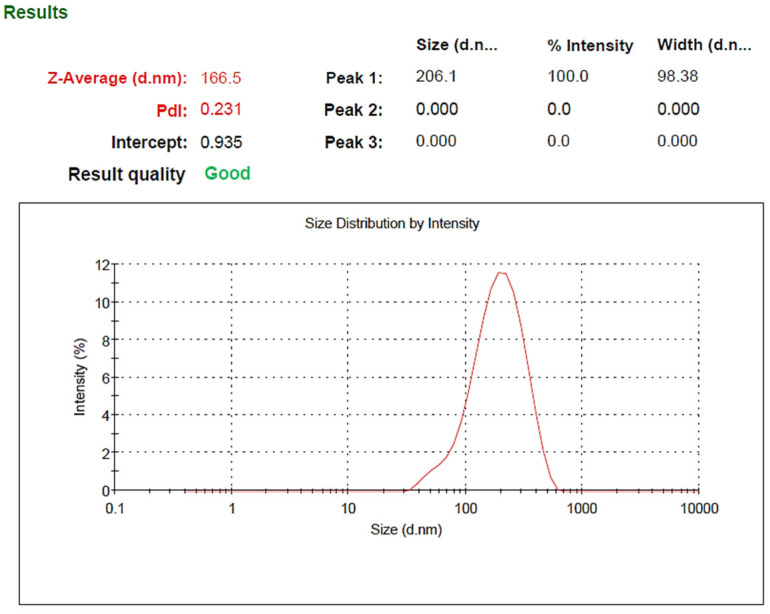
Size distribution of SeN.

**Figure 3:**
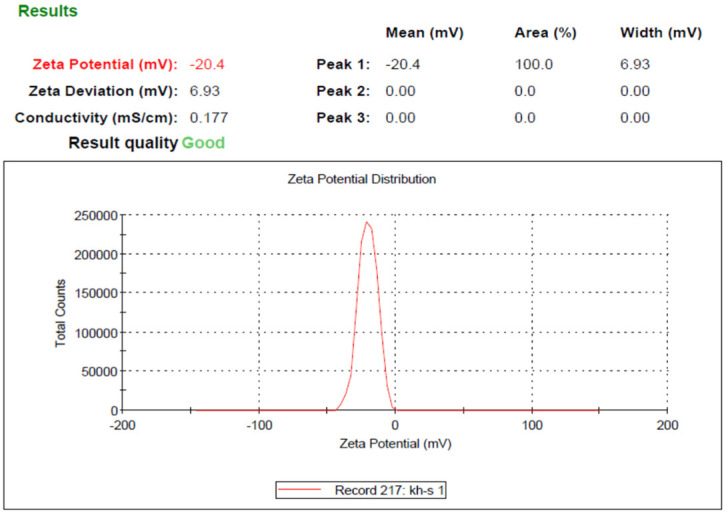
Zeta potential of SeN.

Based on the results obtained from DLS analysis, the nanoparticle size was estimated as 206 nm and the particle size distribution as 0.231, which indicates the uniformity of particle size (Figure 2). Additionally, the hydrodynamic diameter obtained by the DLS method is near the size obtained by the FE-SEM method. The zeta potential of SeN was −20.4, shown in Figure 3.

### 2.3. FTIR Studies

**Figure 4:**
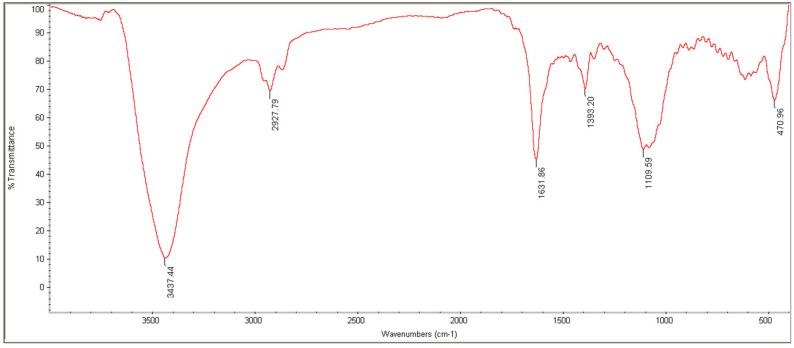
FTIR spectra of SeN.

The results of FTIR are shown in Figure 4. As can be seen, the peak specified in 1105 cm^−1^ is related to Se–O tensile vibrations, and the peak in 1613 and 470 cm^−1^ is related to Se–O bending vibrations. High-intensity bands are observed in 3437 cm^−1^, and 11,631 cm^−1^, which are related to O–H tensile and bending vibrations, respectively, and the peak in 1380 cm^−1^ refers to C–O tensile vibration. The vibrations observed in 2870 cm^−1^ and 2927 cm^−1^ are related to C–H’s symmetric and asymmetric tensile vibrations. 

### 2.4. Investigation of the Appearance of the Prepared Patch

The prepared patch was visually inspected. The prepared patch had a uniform, soft, bubble-free surface, opaque and purple. However, other pharmaceutical analyses were performed on them.

### 2.5. Swelling Index of Prepared Patches

The swelling index investigation of prepared transdermal patches was completed using the abovementioned procedure. The mentioned formula was used to determine the swelling index, and Table 1 contains the results.

### 2.6. Determine the Surface pH of the Prepared Patches

The surface pH of the patches was determined to evaluate the possibility of mucosal stimulation by the patch. Thus, the patch sample was placed in 5 mL of phosphate buffer with pH = 7.4, and the pH was measured at intervals of 2, 4, and 6 h by placing a pH meter electrode on the swollen patch surface. The results showed that all types of made patches had a pH within the normal range of tissue, and the patch in terms of pH did not have any side effects based on tissue irritation (Table 2).

### 2.7. Measuring the Adhesion Strength of the Prepared Patches

The results of measuring the adhesive strength of the patches (Table 3) showed that the prepared ChSeND patches have adhesive strength and can be attached to the surrounding tissues after loading. However, the Ch and ChSeN do not show adhesion strength.

### 2.8. Investigation of Drug Release from Patches Prepared In Vitro

**Figure 5:**
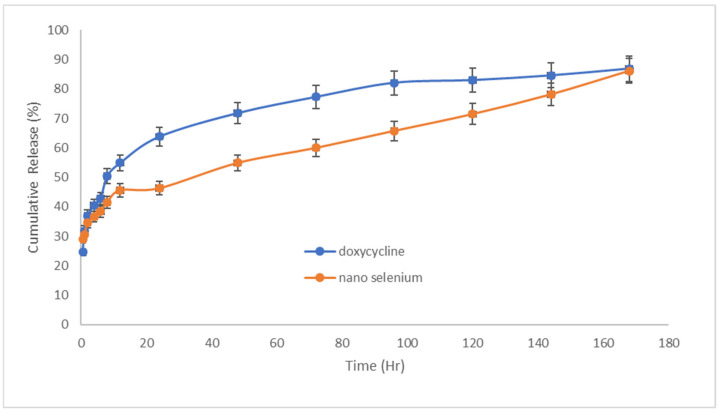
Doxycycline and SeN were released from the transdermal patch at 32 °C and pH = 7.4 (*n* = 3).

The results of drug release from patches can be seen in Figure 5. It was seen that the amount of doxycycline released from the patch in the first 24 h was 63% and the amount of SeN released was 46% during this period, which represents the initial explosive dose of the drugs released. Drug release was studied for up to 7 days. According to the doxycycline release diagram from the patch, it was significantly higher than SeN, which could be related to the particle size of SeN compared to the molecular size of doxycycline. Additionally, both drugs were finally released at 86.4% and 86.2%, doxycycline and SeN, respectively, indicating the patch’s disintegration and the release of all drug content.

### 2.9. Histopathological Examination

**Figure 6:**
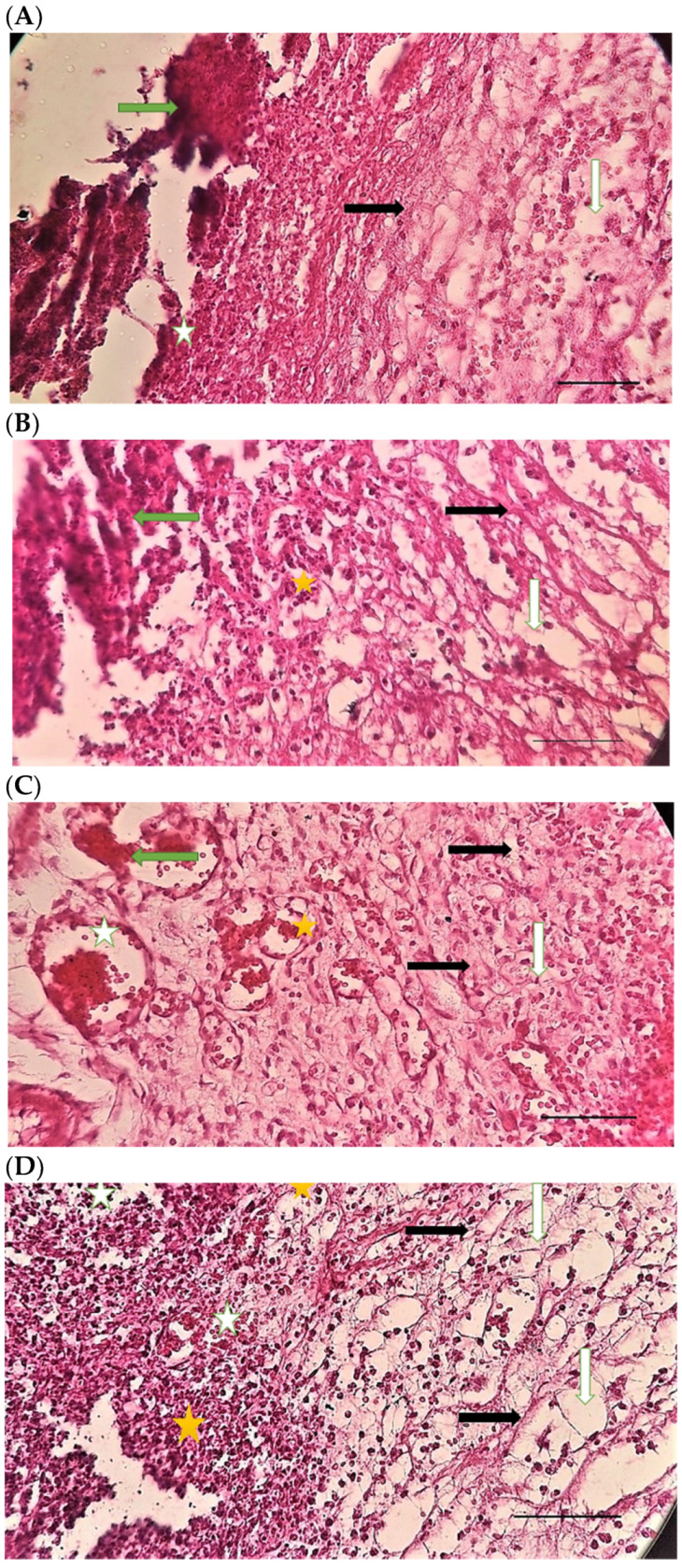
H&E stained microscopic sections (Bar = 100 μm) wound in rats on day 3 of the study. (**A**) Control group; Hemorrhage (white star), lack of collagen fibers formation (black arrow), edema (white arrow). (**B**) Ch group; Edema (white arrow), thin collagen fiber (black arrow), clot (green arrow), inflammation (yellow star). (**C**) ChSeN group; Severe hyperemia (white star), fine collagen fibers (black arrow). (**D**) ChSeND group; Edema (white arrow), hemorrhage (white star), fine collagen fibers (black arrow), inflammation (yellow star).

**Figure 7:**
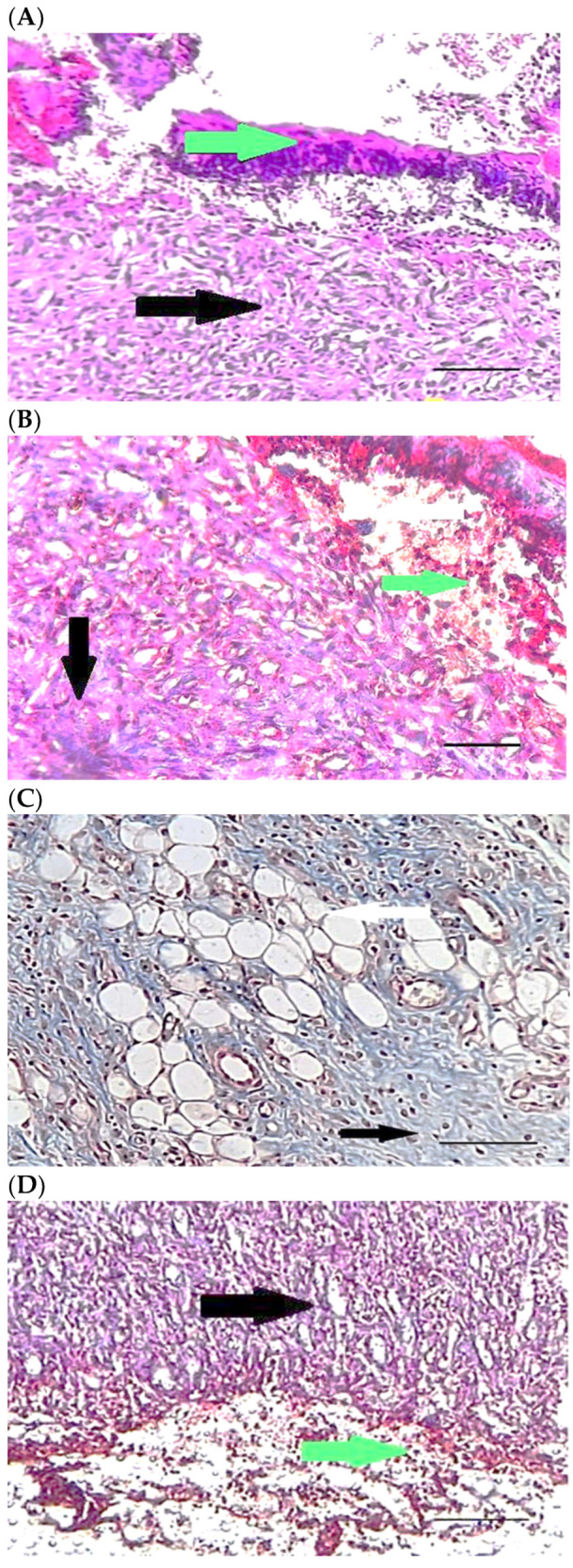
Mason trichrome stained microscopic sections (Bar = 50 μm) wound in rats on day 3 of the study. (**A**) Control group; Clot and hemorrhage (green arrow), fine collagen fiber (black arrow). (**B**) Ch group; Clot (green arrow), fine collagen fiber (black arrow), edema (white arrow). (**C**) ChSeN group; Fine collagen fiber (black arrow), edema (white arrow). (**D**) ChSeND group; Clot and hemorrhage (green arrow), fine collagen fiber (black arrow).

**Figure 8:**
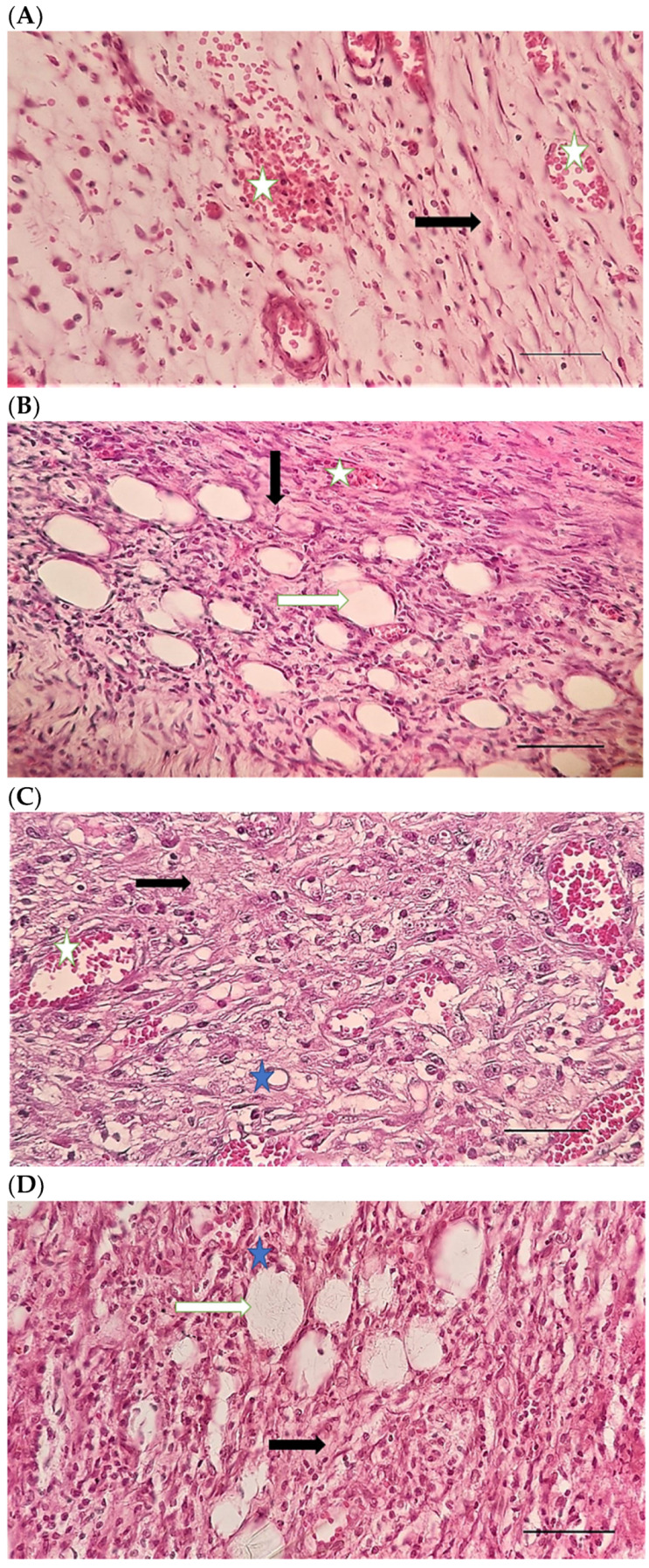
H&E stained microscopic sections (Bar = 100 μm) wound in rats on day 7 of the study. (**A**) Control group; Fine collagen fibers (black arrow), hyperemia and hemorrhage (white star). (**B**) Ch group; Edema (white arrow), fine collagen fibers (black arrow), hyperemia (white star). (**C**) ChSeN group; Fine collagen fibers (black arrow), newly formed vessels (blue star), hyperemia (white star). (**D**) ChSeND group; Edema (white arrow), relatively thick collagen fibers (black arrow).

**Figure 9:**
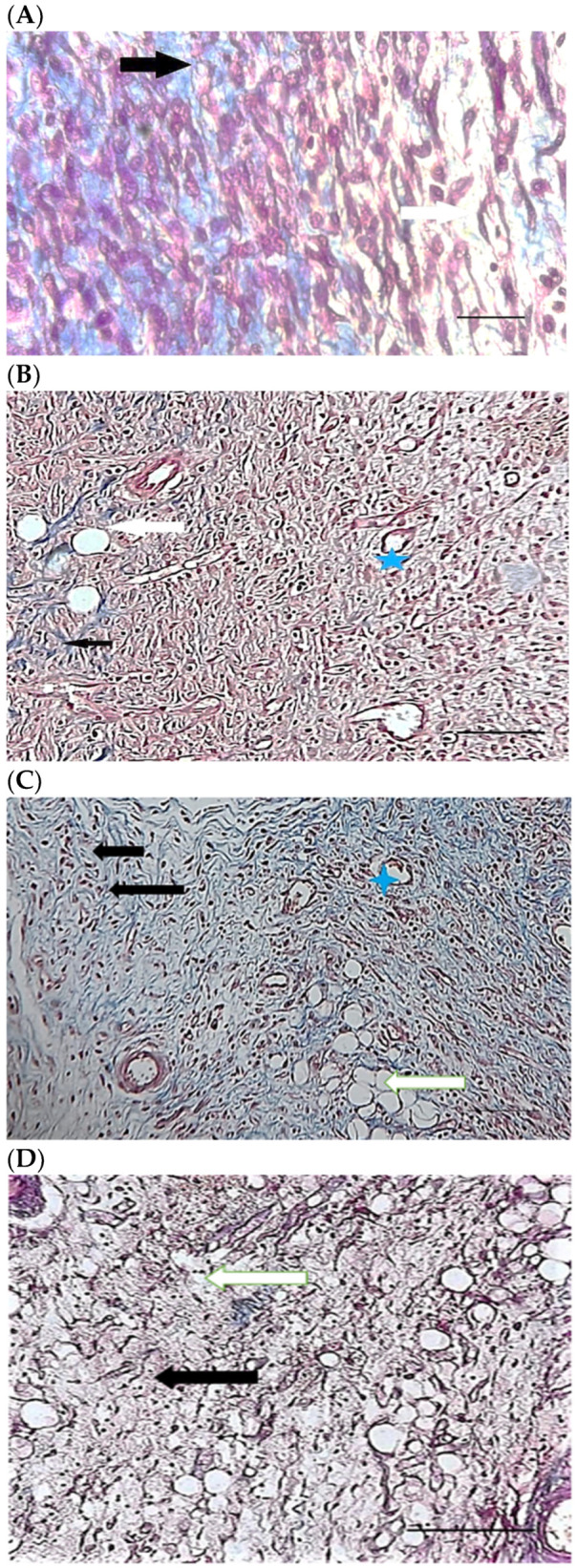
Mason trichrome stained microscopic sections (Bar = 100 μm) wound in rats on day 7 of the study. (**A**) Control group; edema (white edema), scattered and thin collagen fibers (black arrow). (**B**) Ch group; Collagen fibers (black arrow), edema (white arrow), newly formed vessels (blue star). (**C**) ChSeN group; edema (white edema), collagen fibers (black arrow), hyperemia (blue star). (**D**) ChSeND group; edema (white arrow), collagen fibers (black arrow).

**Figure 10:**
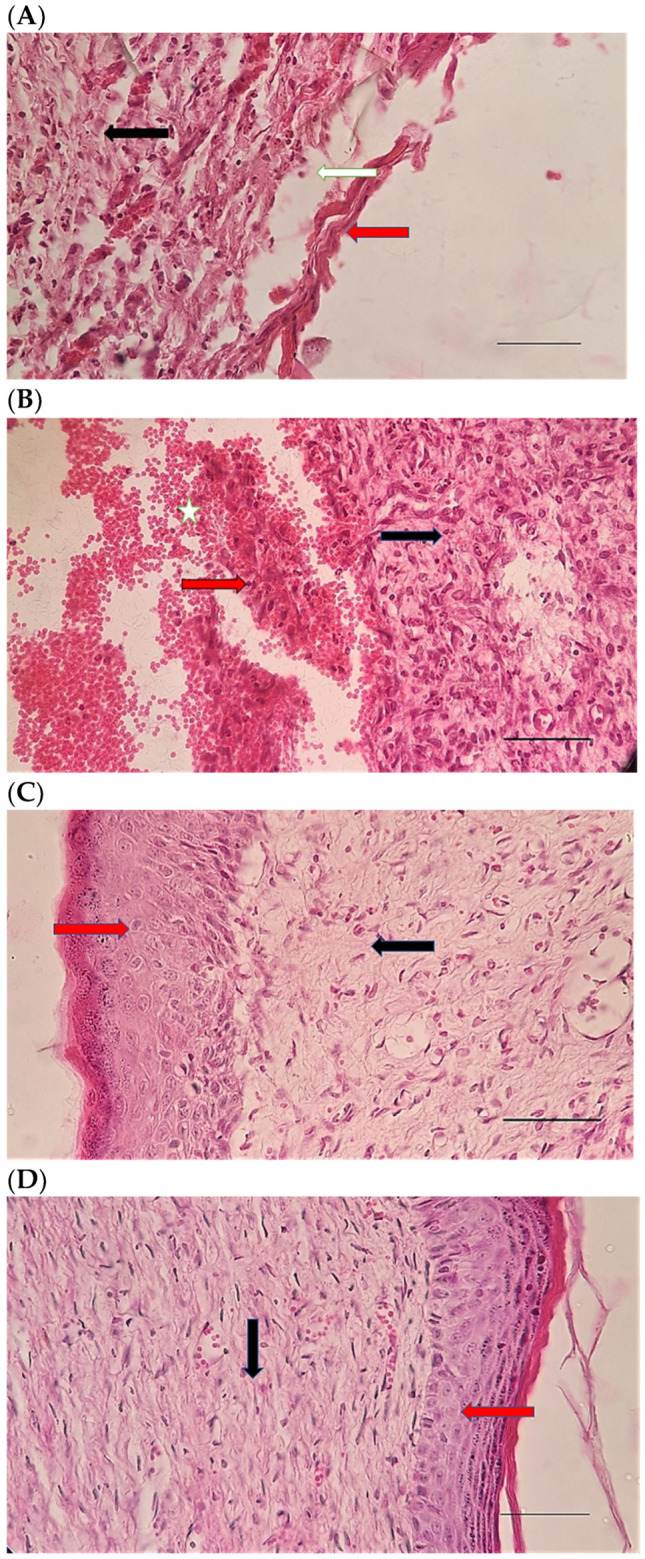
H&E stained microscopic sections (Bar = 100 μm) wound in rats on day 21 of the study. (**A**) Control group; Formation of incoherent collagen fibers (black arrow), incomplete re-epithelialization (red arrow), edema (white arrow). (**B**) Ch group; Fine collagen formation (black arrow), incomplete epithelial tissue formation (red arrow), hemorrhage (white star). (**C**) ChSeN group; Fine collagen formation (black arrow), relatively thick epithelium formation (red arrow). (**D**) ChSeND group; Regular epithelial tissue formation (red arrow), relatively thick collagen fibers (black arrow).

**Figure 11:**
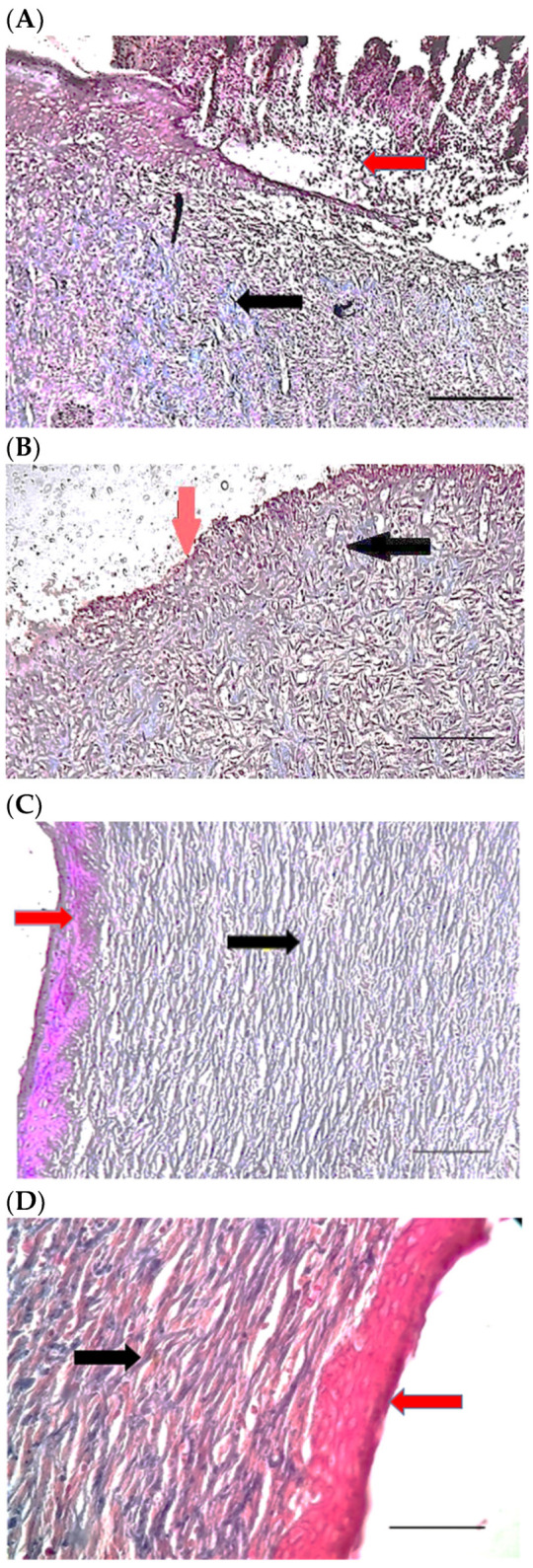
Mason trichrome stained microscopic sections (Bar = 50 μm) wound in rats on day 21 of the study. (**A**) Control group; Incomplete re-epithelialization (red arrow), collagen fibers (black arrow). (**B**) Ch group; Incomplete epithelium formation (red arrow), scattered collagen fibers (black arrow). (**C**) ChSeN group; Re-epithelialization (red arrow), collagen fibers (black arrow). (**D**) ChSeND group; Good re-epithelialization (red arrow), relatively thick collagen fibers (black arrow).

Table 4 compares the evaluated parameters of tissue sections in the different groups. In Figure 6, Figure 7, Figure 8, Figure 9, Figure 10 and Figure 11, tissue sections of different groups have been compared at similar times. On day 3 of the study, no significant difference was observed between the two parameters of collagen formation and re-epithelialization in different groups. However, the hemorrhage parameter was lower in the ChSeND group than in the other groups. Neo-vascularization was higher and more significant in this group than in the other groups. The results showed that the least noticeable inflammation was seen in the ChSeND group, and then the ChSeN group showed less inflammation (Figure 6 and Figure 7). On day 7 of the study, inflammation in the ChSeN and ChSeND groups was significantly less than in the other two groups. In addition, collagen formation was significantly higher in these two groups than in the other two groups. Hemorrhage on the seventh day of the study was higher in the control group than in the other groups. Additionally, Neo-vascularization and re-epithelialization in the ChSeND group were significantly higher than in other groups (Figure 8 and Figure 9). On day 21, the study of tissue inflammation did not show a significant difference between the different groups. Still, hemorrhage was higher in the control and chitosan groups than in the other two groups. The results showed that most neo-vascularization was seen in the ChSeND group and then in the ChSeN group. On this day, maximum collagen formation and re-epithelialization were seen in the ChSeND group. However, re-epithelialization in the ChSeN group was more than in the two groups of chitosan and control (Figure 10 and Figure 11).

Comparison of the mean (± SE) of wound area in different groups showed that on day 3 of the study, there was no significant difference in wound area in different groups (Table 5).

On day 7, the lowest area of the experimental wound belonged to the ChSeND group; however, on day 21, the highest wound area belonged to the control group, and the mean of this measured parameter did not differ significantly in other groups.

Summarizing different results and analyzing the data showed that on day 7 of the study, the best repair belonged to the ChSeND group, but on day 21, the best repair belonged to this group and subsequently to the ChSeN group.

**Table 5:** Comparison of the mean (±SEM) of wound area (mm^2^) in different groups.

	Day 3	Day 7	Day 21
**Control**	249.43 ± 22.02 ^a^	148.03 ± 14.33 ^a^	7.5 ± 2.51 ^a^
**Ch**	245.45 ± 26.27 ^a^	146.3 ± 14.7 ^a^	1.7 ± 0.5 ^b^
**ChSeN**	188.06 ± 106.16 ^a^	135.88 ± 21.58 ^a^	1.52 ± 0.92 ^b^
**ChSeND**	169.4 ± 76.07 ^a^	100.71 ± 11.23 ^b^	1.35 ± 0.63 ^b^

The presence of different letters in each column indicates a significant difference. The significance level was considered *p* ˂ 0.05.

## 3. Materials and Methods

### 3.1. Materials

Selenium dioxide, ascorbic acid, low molecular chitosan and propylene glycol were purchased from Merck (Darmstadt, Germany). Moreover, doxycycline was obtained from Razak Pharma (Razak Pharma Co., Karaj, Iran).

All the stages of design and conduct of the present study were reviewed and approved by the Council of the Department of Veterinary Clinical Sciences of Shahrekord University (54739-1399.10.27).

#### 3.1.1. Preparation of Selenium Nanoparticles Loaded by Doxycycline

First, 500 mL of selenium dioxide solution was made at 1 mM concentration and was left with high-speed stirring. In the next step, 50 mL of 44 mM ascorbic acid solution was added dropwise to the above solution to form selenium nanoparticles (SeN). The formed nanoparticles were separated by centrifugation and dried. To prepare drug-loaded nanoparticles, 15 mg of selenium nanoparticles and 15 mg of doxycycline were dispersed in 5 mL of ethanol and stirred for 24 h. The last suspension was dried in the oven at 40 °C to obtain the final particles as SeND.

#### 3.1.2. Investigation of Prepared Nanoparticles

Particle size and zeta potential of SeN were investigated using a ZetaSizer (Mastersizer 2000; Malvern Instruments, Malvern, Worcestershire, UK), and morphological properties were investigated using a FE-SEM microscope (FE-SEM, Tescan/Mira, Brno, Czech Republic). Moreover, the prepared nanoparticles were studied by infrared (IR) spectroscopy recorded on a Nicolet Magna IR-550 (Madison, WI, USA) using KBr pellets.

#### 3.1.3. Preparation of Transdermal Patch

First, 100 mg of chitosan was dispersed in 20 mL of 1% acetic acid and stirred for 2 h to obtain a homogeneous solution. After, 0.2 mL of propylene glycol and 1.2 mg of SeND were added to the previous solution. This new mixture was stirred for 24 h at room temperature. The final mixture was poured into 20 circular 1 × 1 cm^2^ diameter molds and they were allowed to dry at room temperature overnight. The dried patches were named as ChSeND. Moreover, other groups of patches were prepared with the same process as ChSeND but instead of SeND, 0.6 mg SeN (14) and zero amount of nanoparticle were added to ChSeN and Ch transdermal patches, respectively. Final prepared patches were checked visually for defects or bubbles. The fault-free patches were taken, wrapped in aluminum foil, and stored in a glass container at room temperature for later steps.

#### 3.1.4. Determining the Thickness and Swelling Index of the Prepared Patch

After determining the patch’s initial weight (W_0_), samples were placed on a 2% agar plate’s surface and heated to 32 °C to measure the patches’ swelling. The patches were removed from the oven at regular intervals to remove any extra water off the surface with filter paper, weigh the swollen patch again (Wt), and use the below formula to obtain the swelling index of the patch.

$$swelling\;index=\frac{\;\mathrm{Wt}-W0}{W0}*100$$

#### 3.1.5. Determine the Surface pH of the Prepared Patch

The patch’s surface pH was measured to assess the likelihood of the patch stimulating tissue. The patch samples were placed in 5 mL phosphate buffer at 7.4 pH. The pH was then monitored using a digital pH meter (Metrohm 827, Herisau, Switzerland) at 2, 4, and 6 h intervals by inserting an electrode on the patch’s surface. Before each measurement, the pH meter was calibrated with reference buffers, and the mean was computed after three repetitions.

#### 3.1.6. Measurement of In Vitro Adhesion Strength of the Prepared Patch

**Figure 12:**
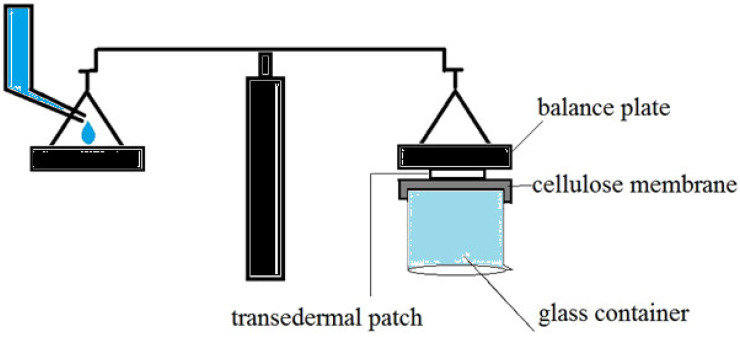
In vitro techniques for measurement of adhesion strength of the transdermal patch.

The adhesion strength of a patch has been evaluated using various in vitro techniques. To determine the lowest adhesion force formed between the patch and the mucosa, almost all of these approaches assessed the force required to detach a patch from a smooth surface. First, a 1*2 cm^2^ piece of cellulose membrane was glued to the back of a glass container and hydrated with distilled water, as shown in Figure 12. After that, one of the prepared patches attached to the balance behind the plate was pressed against the surface of the cellulose membrane for one minute. The water was then poured into another plate of balance drop by drop at a rate of 3 mL/min. To calculate the adhesion strength, the time the patch was removed from the surface of the cellulose membrane was recorded. This experiment was repeated three times, with the average result reported.

#### 3.1.7. Investigation of SeN and Doxycycline Release from the Prepared Patch

Transdermal in vitro release studies were performed using a cell Franz diffusion with a 20 mL receptor chamber capacity. A cellulose acetate membrane (pore size 0.45 μm) separated the recipient and donor part of the Franz cell. Aluminum foil was used to cover the prepared patch before placing it on the cellulose acetate membrane. A tiny magnet was placed inside the receptor chamber cell after filling it with phosphate buffer (pH: 7.4). The receptor chamber cell’s solution was agitated steadily and kept at a temperature of 32 ± 0.5 °C. At the same time, the entire assembly was positioned on a hot magnetic stirrer. Samples were obtained, and an equal volume of phosphate buffer was supplied at intervals of 1, 2, 3, 4, 6, 12, 24, 48, 72, 96, 120, 144 and 168 h. To ascertain the drug content in each sample, spectrophotometry was used to evaluate it. The amount of selenium and doxycycline were determined at 570 and 240 nm lambda max as reference methods [15,16].

### 3.2. Animals and Excisional Wounding

Eighty adult two-month-old female Wistar rats weighing 250–270 g were purchased and kept for two weeks in the Rodent Center of our institute under standard feeding and care conditions. After adopting the animals to the new environment and confirming their clinical, behavioral and nutritional health, they were randomly divided into four equal groups. (A) The control group who had skin defects and did not receive any therapeutic intervention; (B) the chitosan group in which a Ch (chitosan transdermal patch) was placed on the skin defects; (C) the ChSeN (chitosan-selenium nanoparticle) group in which the chitosan transdermal patch loaded with selenium nanoparticles was placed on the skin defects; (D) the ChSeND (chitosan-selenium nanoparticle-doxycycline) group in which the chitosan transdermal patch containing doxycycline and selenium nanoparticle was applied to the skin defects;

Rats were anesthetized by intraperitoneal injection of ketamine (70 mg/kg; Ketamine 5%; Alfasan; Woerden, The Netherlands) and xylazine (10 mg/kg; xylazine 2%; Alfasan; Woerden, The Netherlands) [17], and placed in a sternal position, and the hairs on their back were clipped and the skin was aseptically prepared for surgery. Then, a square excision wound was made by cutting away the full thickness of the skin (1.5 cm × 1.5 cm) to create skin defects on the back of all animals. The control group had only the usual wound dressing without intervention. Still, in other groups, different transdermal patches (depending on the kind of group) were placed on the wounds and in all rats, a 5-day dressing was used to cover the patches and wounds.

The rats were then transferred to their cages for daily animal care (including wound and dressing preservation, feeding and access to water, etc.) and environmental management (including feeding and litter replacement) and providing the standard temperature and humidity and so on.

### 3.3. Histological Examination

Time 3, 7, and 21 were considered as the time to collect tissue samples after skin defects were induced. At any time, seven rats from each group were used for sampling and measuring the wound area. In addition to measuring the wound area (mm^2^) at different times, rats were anesthetized by intraperitoneal injection of a combination of anesthetics and skin samples were taken for histological examination. For this purpose, a thick piece of the skin tissue was removed from the edges of the wound, including healthy and damaged skin, with a scalpel and sent to a histology laboratory in a container containing 10% formalin buffer.

Following routine suturing of the affected skin and recovery of the animals, the rats were transferred to their previous location, the Rodent Center of the institute. Following the preparation of three parallel sections of each sample, H&E staining (for the evaluation of inflammation, hemorrhage, collagen fiber orientation, neo-vascularization, and re-epithelialization) and mason trichrome staining (to identify collagen fibers) were performed.

### 3.4. Statistical Analysis

The median of tissue parameters between different groups, after scoring of evaluated parameters [based on the severity of the event: 0 (absence), (1) weak, (2) moderate, (3) high], was compared to a Kruskal–Wallis statistical test with a significance level of *p* ˂ 0.05. In addition, the mean (±SEM) wound area in different groups was compared using a one-way analysis of variance (at a significance level of *p* < 0.05).

## 4. Discussion

Transdermal patches are now routinely used for medication administration. Skin patches have been used for various purposes, including medicine delivery to alleviate pain, repair wounds, treat aphthous ulcers, and manage nausea. Dermal medication administration has a lengthy history of treating local or systemic problems [18,19]. The current investigation found that the manufactured transdermal preparation patch had an acceptable appearance, disintegration time, thickness and swelling, surface pH, film adhesion, and in vitro release characteristics. The results indicate that this patch can be examined and utilized in treating various skin lesions, such as skin wounds, in the following phases.

Monodispersed SeN with a particle size of around 200 nm was successfully created using the precipitation method. SeN indicated a spherical shape and restricted size distribution, as shown in Figure 1. According to various research, spherical SeN in the size range of 30–200 nm can be produced via chemical synthesis of SeN [20]. Additionally, the Fe-SEM values for the SeN were supported by DLS results. The Zeta potential of SeN was found to be −20 mV, as shown in Figure 3, which is supported by a previous study. The outcomes of FTIR experiments on SeN are displayed in Figure 4.

Nevertheless, the peaks C=O and Se–O were found. These results demonstrate that some starting material, such as SeO2 and ascorbic acid, has been trapped by SeN [21]. There are many reasons for using chitosan in skin wound repair, including its antibacterial [22], anti-inflammatory effects [23], and induction of skin regeneration [22]. In addition, the good water absorption and retention properties, biodegradability and biocompatibility of chitosan should also be considered [24]. In addition, it has been reported that chitosan can be used in wound dressings because it has good hydrophilicity and is an exudate adsorbent [25] and maintains wound moisture [26]. Chitosan wound dressings are a physical barrier against microbial contamination of wounds [25,27].

Although the results of the present study did not indicate a significant difference between the measured tissue parameters between the control and Ch groups, it was found that on day 21 of the study, the wound area in the chitosan group was significantly reduced compared to the control group. Additionally, the addition of compounds that are effective in the healing of skin wounds to chitosan scaffolding has been reported in various studies [24]. These include the addition of metallic nanoparticles such as gold nanoparticles, copper nanoparticles, silver nanoparticles, zinc nanoparticles, and selenium nanoparticles, which have been considered in various studies in the wound healing process [28,29].

In the present study, the loading of doxycycline and selenium nanoparticles on the chitosan scaffold was considered for synthesizing a controlled-release system, and their local application in skin wounds was investigated. It was observed that adding selenium nanoparticles to the chitosan scaffold improved the therapeutic effects of the chitosan transdermal patch. The use of selenium nanoparticles has been reported frequently due to their strong and long-acting antioxidant effects. These nanoparticles can inhibit bacterial growth [30]. The study of Abbaszadeh et al. (2019) reported that angiogenesis and cell infiltration (population of polymorphonuclear and mononuclear leukocytes) in the application of chitosan biofilm containing selenium nanoparticles on skin wounds infected with Staphylococcus aureus were higher than the control group [31].

Similarly, in the study of Rostami et al. (2018), it was stated that the topical application of chitosan film containing selenium nanoparticles in full-thickness rats wounds increases angiogenesis, collagen formation, and the number of fibroblasts, and wound healing improves [32]. Similar to the results of the present study, which showed the usefulness of selenium nanoparticles in wound healing and faster reduction of the wound area, it is stated that selenium nanoparticles increase the level of vascular endothelial growth factor (VEGF) and collagenase 1 and decrease the level of nitric oxide [33]. VEGF regulates angiogenesis and stimulates the formation of new blood vessels by stimulating endothelial cell proliferation, migration, differentiation, and survival [34]. At the same time, nitric oxide (which plays an important role in all phases of wound healing) increases nitroxidative stress [35]. The present study’s results showed that adding doxycycline to the chitosan system containing selenium nanoparticles (ChSeND group) provides the best repair among different groups.

Doxycycline, as a tetracycline, is involved in inflammation, new vascular formation, and tissue regeneration by inhibiting metalloproteases [8]. Doxycycline inhibits reactive oxygen species and thus prevents the conversion of proMMP to active matrix metalloproteinases [9,10]. In addition, it can inhibit protein synthesis by binding to bacterial ribosomes [8]. Therefore, with the mentioned mechanisms, doxycycline had a synergistic effect in the experimental wound healing of the present study. Similarly, the study by Hedayatyanfard et al. (2020) showed that local application of chitosan-polyvinyl alcohol containing doxycycline positively affected the healing of diabetic wounds in rats. This dressing decreased inflammation and increased collagen levels, the number of fibroblasts, and vascularization [36]. In addition, the non-cytotoxicity of chitosan-doxycycline-collagen three-layer nanofiber dressing against keratinocyte cells has been reported [11].

## 5. Conclusions

The results of the present study, by previous studies, confirmed the use of chitosan as a suitable scaffold in transdermal patches. In addition, the evaluation of wound area and histological parameters confirmed the good effects of selenium nanoparticles and doxycycline in wound healing. However, the evaluation of the ChSeND group showed that the simultaneous presence of selenium nanoparticles and doxycycline provides the best repair compared to the control group and the chitosan and ChSeN groups. In addition, the effect of this combination on the healing of skin wounds in diabetic models can be investigated in future studies, and more evaluations such as measuring tissue biomarkers and reepithelialization are also suggested.

## Figures and Tables

**Table 1:** Swelling index of different patches.

Patch Type	W_0_ (mg)	W_t_ (mg)	Swelling Index
Ch	0.0041	0.0123	200.00
ChSeN	0.0051	0.0133	536.54
ChSeND	0.0095	0.2037	2044.21

**Table 2:** Surface pH of different patches at determined times.

Patch Type	Time/h		
	2	4	6
Ch	7.37	7.35	7.33
ChSeN	7.28	7.23	7.23
ChSeND	6.98	7	6.99

**Table 3:** Adhesion strength of different patches.

Patch Type	Repetition/Time (s)			
	1	2	3	Mean
Ch	0	0	0	0
ChSeN	0	0	0	0
ChSeND	85	103	86	91.33 ± 10.11

**Table 4:** Comparison of the median of various tissue parameters between different groups.

	Day 3					Day 7					Day 21				
	Inflammation	Hemorrhage	Neo-Vascularization	Collagen formation	Re-Epithelialization	Inflammation	Hemorrhage	Neo-Vascularization	Collagen Formation	Re-Epithelialization	Inflammation	Hemorrhage	Neo-Vascularization	Collagen Formation	Re-Epithelialization
**Control**	3 (2–3) ^a^	3 (2–3) _a_	0 (0–1) ^a^	0 (0–1) ^a^	0 (0–0) ^a^	3 (1–3) ^a^	2 (1–2) ^a^	1 (0–1) ^a^	1 (0–1) ^a^	0 (0–1) ^a^	1 (0–1) ^a^	1 (0–1) ^a^	0 (0–1) ^a^	2 (0–2) ^a^	1 (0–1) ^a^
**Ch**	2 (2–3) ^a^	3 (1–3) ^a^	1 (0–1) ^a^	1 (0–1) ^a^	0 (0–0) ^a^	3 (1–3) ^a^	1 (0–1) ^b^	1 (0–1) ^a^	1 (1–1) ^a^	0 (0–1) ^a^	1 (0–1) ^a^	1 (0–1) ^a^	0 (0–1) ^a^	2 (1–2) ^a^	1 (1–1) ^a^
**ChSeN**	2 (2–2) ^b^	3 (1–3) ^a^	1 (0–1) ^a^	1 (0–1) ^a^	0 (0–0) ^a^	1 (0–2) ^b^	1 (0–1) ^b^	1 (0–1) ^a^	2 (0–2) ^b^	0 (0–1) ^a^	0 (0–1) ^a^	0 (0–0) ^b^	1 (0–1) ^a^	2 (1–2) ^a^	2 (1–2) ^b^
**ChSeND**	1 (1–2) ^c^	2 (1–3) ^b^	2 (0–2) ^b^	1 (1–1) ^a^	0 (0–0) ^a^	1 (0–1) ^b^	0 (0–1) ^b^	3 (1–3) ^b^	2 (1–2) ^b^	1 (1–2) ^b^	0 (0–0) ^a^	0 (0–0) ^b^	1 (0–1) ^a^	3 (2–3) ^b^	3 (2–3) ^c^

Different letters in each column indicate a significant difference.

## Data Availability

Data is contained within the article.
